# The Use of Contrast-Enhanced Sonography for Therapy Monitoring of Metastatic Lymph Nodes: A Systematic Review

**DOI:** 10.3390/curroncol30070494

**Published:** 2023-07-16

**Authors:** Maximilian Rink, Ernst-Michael Jung, Julian Künzel

**Affiliations:** 1 Department of Otorhinolaryngology, Head and Neck Surgery, University Hospital of Regensburg, 93053 Regensburg, Germany; 2 Department of Radiology, University Hospital of Regensburg, 93053 Regensburg, Germany

**Keywords:** contrast-enhanced ultrasound, CEUS, therapy monitoring, solid carcinoma, head and neck squamous carcinoma, lymph node metastasis

## Abstract

Metastatic cervical lymph nodes are a frequent finding in head and neck squamous cell carcinoma (HNSCC). If a non-surgical approach is primarily chosen, a therapy response evaluation of the primary tumor and the affected lymph nodes is necessary in the follow-up. Supplementary contrast-enhanced ultrasound (CEUS) can be used to precisely visualize the microcirculation of the target lesion in the neck, whereby malignant and benign findings differ in their uptake behavior. The same applies to many other solid tumors. For various tumor entities, it has already been shown that therapy monitoring is possible through regular contrast-enhanced sonography of the primary tumor or the affected lymph nodes. Thus, in some cases, maybe in the future, a change in therapy strategy can be achieved at an early stage in the case of non-response or, in the case of therapy success, a de-escalation of subsequent (surgical) measures can be achieved. In this paper, a systematic review of the available studies and a discussion of the potential of therapy monitoring by means of CEUS in HNSCC are presented.

## 1. Introduction

Head and neck squamous cell carcinoma (HNSCC) is one of the most common tumor entities worldwide [1]. In addition to the use of alcohol [2] and smoking [3], viral infections with the Epstein–Barr virus (EBV) [4] and cancerogenic types of the human papilloma virus (HPV) [5] are the main risk factors. In advanced tumors, lymph node metastases are frequent [6].

The therapy should always be adapted to the individual case. There are surgical and chemoradiation concepts for the treatment of HNSCC [7], which can also be combined in advanced cases to improve the outcome of the patient [8]. Immunotherapy has become increasingly available in the neoadjuvant and palliative setting (recurrent and metastatic HNSCC), as well as in combination with radiotherapy within clinical trials [9,10,11,12,13,14].

If a primarily non-surgical treatment is chosen, the evaluation of the primary tumor and possible affected lymph nodes is necessary for the evaluation of the response to therapy. Positron emission computed tomography (PET-CT) plays a major role in this [15]. However, PET-CT cannot always distinguish between residual vital tumor tissue and inflammatory activity. False positive findings seem to occur more frequently after radiochemotherapy than after radiation alone [16]. One possibility in the case of inconclusive findings is a further PET-CT in the short-term course [17]. However, this is associated with additional radiation exposure for the patients and is also cost intensive. Another limitation of PET-CT is the time of application. In order to keep the rate of false positive findings as low as possible, the first scan is recommended about 12 weeks after the end of therapy [15]. Early and repeated monitoring regarding the success of an ongoing therapy using PET-CT is therefore neither sensible nor justifiable from a radiation protection perspective.

Fundamental B-scan sonography also plays a major role in follow-up imaging of HNSCCs [18]. The significance is limited by the lack of generally accepted criteria classifying a lymph node as malignant. This is made even more difficult in follow-up care because, for example, the sonographic appearance of malignant lymph nodes is altered by radiation [19]. Here, the performance of a (supplementary) contrast-enhanced sonography (CEUS) could contribute to an improved monitoring of the therapy success. This involves the application of gas-filled microbubbles, usually intravenously. In this way, real-time imaging of the microcirculation is possible. CEUS is an easy to perform, safe procedure with low risks. This is also true in pediatric population. Serious side effects like allergic reactions are rare [20]. Nevertheless, appropriate equipment should be available for those emergencies and the investigators should be trained in their management on a regular basis. Minor possible side effects could be headaches, chest pain or nausea. If the known contraindications (e.g., pulmonal hypertonia, pregnancy, persisting foramen ovale) are respected, it can also be performed repeatedly. There is no radiation exposure for the patient. Different to contrast media used for CT, the use of contrast agent for CEUS is not limited by the renal and thyroid function of the patients. Blood tests prior to performance are not necessary [21]. In conclusion, CEUS is a promising imaging tool for therapy monitoring, regarding its beneficent safety profile, the easy use and the low costs compared to other imaging modalities. Of course, this is only possible if the lesion of interest can be displayed in B-scan sonography.

CEUS for non-liver indications is published by the European Federation of Societies for Ultrasound in Medicine and Biology (EFSUMB) guidelines, normally performed using a bolus injection of 1–2.4 mL SonoVue as contrast agent. It is crucial to use a low mechanical index (<0.16) to avoid destruction of the microbubbles [21]. As part of the evaluation, a distinction must be made between qualitative and quantitative analyses. In the quantitative analysis, objective parameters are calculated with the help of special software; in the qualitative analysis, the examiner subjectively rates, for example, the homogeneity of the contrast medium uptake of the target structure. It is crucial that the operators have sufficient expertise in performing and analyzing CEUS.

Various studies have already shown differences between benign and metastatic lymph nodes in the examination with CEUS [22,23,24]. Characteristics of metastatic lymph nodes are, among other things, a heterogeneous accumulation of the contrast medium within the lymph node [25], a centripetal (directed from the periphery to the center) spread of the contrast medium [26,27] as well as a more rapid uptake of the contrast medium [22,24,27], represented by a shortened time to peak (TTP). However, whether CEUS allows a reliable differential diagnosis between benign and malignant lymph nodes is still controversial. For example, Lerchbaumer et al. found no significant differences in quantitative assessment [28].

Previous studies have shown that CEUS is suitable for assessing the success of systemic lymphoma therapy [29]. The aim of the present study was therefore to conduct a systematic literature review to examine the existing evidence for contrast medium ultrasound (CEUS) of superficial lymph node metastases of solid tumors under radiation, chemotherapy or immunotherapy as a method for therapy evaluation. It is necessary to keep in mind that this represents an “off-label use” of CEUS. The patients need to be informed of this fact.

## 2. Materials and Methods

The inclusion and exclusion criteria were defined using the PICOS acronym (see Table 1). Only articles in English or German were included.

**Figure 1:**
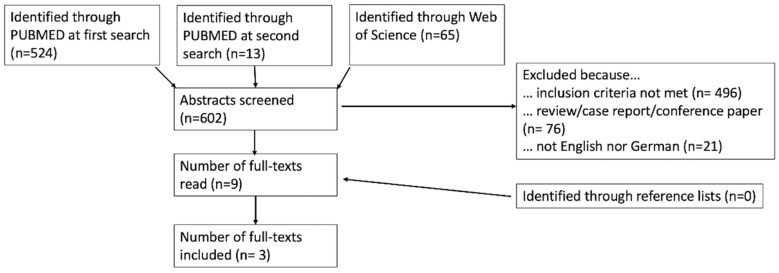
Presentation of the literature research.

We performed a systematic review. A literature search was carried out in PUBMED/Medline and in Web of Science. The search contained a combination of the following terms: “CEUS OR contrast enhanced ultrasound OR contrast-enhanced ultrasound AND lymph nodes OR lymphatic AND therapy response OR therapy evaluation OR therapy monitoring”. We also searched the source indexes of the full texts we read. The search process is shown in Figure 1, based on the PRISMA guidelines [30]. The PUBMED search was first performed on 25 February 2023, and the Web of Science search was first performed on 25 June 2023. Both searches were conducted a second time on 5 July 2023 to consider possible new publications. This identified 13 new abstracts in PUBMED (zero in Web of Science); none of them met the inclusion criteria in our review.

Since metastases of solid tumors and lymphomas appear different in CEUS [31], studies concerning therapy monitoring of lymphomas were not considered. Since the present study examines the monitoring of lymph node metastases, studies that examine the success of therapy based on changes in the primary tumor or distant metastases in other organs (e.g., liver metastases in nasopharyngeal carcinoma) in CEUS were not included [32,33,34,35,36,37,38,39,40,41].

## 3. Results

Overall, the titles and abstracts of 602 studies were screened. A total of nine full texts were read and considered for inclusion. Three of these were excluded because the treatment response of the primary tumor was assessed with CEUS [32,33,42]. Two of the others addressed the finding of a sentinel lymph node following neoadjuvant therapy in breast cancer [43] and conversion surgery in gastric cancer [44] and were therefore excluded. In the paper of McCarville et al., only one of the lesions studied was a lymph node [45]. In summary, only three papers matched all our inclusion criteria.

In addition to the database search, the source lists of the nine read full texts were searched for additional studies, but no further work was found for inclusion. The entire search process is shown in Figure 1.

Table 2 and Table 3 provide an overview of the included studies and their results. SonoVue^®^ (Bracco, Milan, Italy) was used as a contrast agent in all studies.

Ye et al. studied patients with nasopharyngeal carcinoma and histologically confirmed lymph node metastases. All their patients received radiotherapy; most of them also received an additional systemic therapy (52 additional platinum-based chemotherapy, 10 additional nimotuzumab). The examinations were performed before the start of treatment and during the 5th radiation session. Quantitative analysis was performed using Qontrast software (Bracco, Milan, Italy). This tool can be used to analyze the examinations previously made and to calculate quantitative parameters. Magnetic resonance imaging (MRI) was performed as a reference procedure before and one month after therapy to assess the success of therapy. The authors were able to show that peak intensity (PI) values or their alteration during therapy correlated significantly with the response to therapy. PI is a measure of perfusion intensity. The PI values decreased in all patients during therapy, but the PI was significantly higher in patients with a complete response during therapy than in patients with a partial response. The authors discuss that improved blood flow and thereby possibly improved oxygenation in the early phase of therapy benefits the outcome. To improve standardization, the change in PI (PI _before therapy_ − PI _under therapy_) and a corresponding quotient (PI _under therapy_/PI _before therapy_) were also calculated. Again, there were significant differences between patients with a complete and partial response. For time to peak (TTP), a measure of perfusion speed, such a correlation could not be demonstrated. The best prediction of treatment success was achieved by PI _under therapy_ and the quotient mentioned above [40].

Chen et al. reported comparable results in their study with histologically confirmed advanced nasopharyngeal carcinomas. Before the start of radiochemotherapy, the patients received 14 days of therapy with famitinib. Before (day 0), during (day 8) and after (day 15) therapy, CEUS of the lymph nodes was performed. Here, too, there was a significant correlation between the percentage change in PI and the response to therapy. There were additional parameters showing significant correlation (see Table 3). Patients with minor changes in the perfusion parameters after one week showed a higher risk of progression during the course of therapy [39]. It is important to note that in the study by Chen et al., the assessment of response to therapy by CEUS was only one objective of the study. Additionally, the use of famitinib in nasopharyngeal carcinoma was investigated as a phase 1, dose-escalation study [39].

Han et al. examined the axillary lymph nodes in patients with breast cancer before and after neoadjuvant chemotherapy/endocrine therapy by using the B-Mode ultrasound in combination with CEUS. The histological results of the subsequent surgery or of the samples taken before the initiation of therapy served as the reference for determining the dignity. Quantitative parameters were not analyzed in this work, but a qualitative assessment concerning the pattern of the contrast agent was significantly better at predicting therapeutic success on the axillary lymph nodes than purely conventional B-scan sonography. According to the authors, quantitative analysis was not used because it is not conducive in clinical practice in their opinion [41].

## 4. Discussion

All included studies conclude that CEUS is a promising tool for the early assessment of treatment response in metastatic lymph nodes. In other studies, CEUS could already be used to evaluate the therapy response through repeated examinations of the primary tumor [34,35,36]. For HNSCC in particular, the possibility of assessing the lymph nodes instead of the primaries is important, since unlike, for example, in breast cancer, the primaries cannot always be visualized well in some locations (e.g., hypopharynx/larynx).

Considering the quantitative parameters analyzed in the studies by Ye at al. and Chen et al., it is notable that the PI or the change in PI could be worked out in both studies as a significant prognostic factor for the therapy response. In a study by Xin et al. in which the success of chemotherapy in lymphoma treatment was observed by CEUS of the affected lymph nodes, the PI and the change in PI were also shown to be significant parameters in addition to the area under the curve (AUC) as a measure of the perfusion volume. Studies focusing on the primary tumor also showed significant results for PI and its change in the course of therapy for the prediction of the therapy response [32,34]. In addition to assessing the response to therapy, the PI may also serve as a general indicator for the differential diagnosis between malignant and benign lymph nodes [23]. Taking into account the data available to date, PI represents a promising parameter for monitoring therapy using CEUS.

What is very interesting about the results of Han et al. is that the authors decided not to analyze quantitative parameters, but CEUS also appeared to be promising using a purely qualitative analysis [41]. As the authors mentioned, quantitative analysis is significantly more time-consuming and partly requires additional software. The possibility of monitoring therapy by means of a solely qualitative analysis would facilitate the use of CEUS in this context considerably.

One of the major advantages of CEUS as a tool for therapy monitoring could be the early assessment reported by Chen et al. and Ye et al. in consideration of the available data [39,40] and the possibility of repeated evaluation. This could enable the clinician to change an unsuccessful treatment in an early phase and maybe also be the basis of a de-escalation strategy, limiting the side effects coming together with therapy. By using PET-CT, this kind of early and repeated evaluation is not possible, due to radiation exposure, cost effectiveness and the already mentioned risk of false positive findings [16,17].

In addition to imaging, laboratory approaches to monitoring treatment response (e.g., circulating tumor DNA (ctDNA) or tumor exosomes) in HNSCC also exist [46,47,48,49]. However, these always assess the overall response, not specifically the lymph nodes. Currently, laboratory methods have not become a standard in clinical routine.

**Figure 2:**
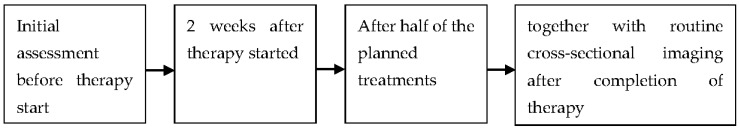
Suggestions for reasonable times for contrast-enhanced ultrasound in the context of therapy monitoring.

All of the limited number of studies conducted so far have examined small groups of patients. Of course, this limits the generalizability of the results. Nevertheless, we think that our strict exclusion criteria are necessary because we wanted to focus on metastatic lymph nodes. Lymphomatous lymph nodes differ in their sonographic behavior, so these papers needed to be excluded. The same is true regarding research about sonographic monitoring of the primary tumor. In addition, the included studies differ in the exact performance of the CEUS examination and different parameters are evaluated. This makes direct comparability difficult. To further establish CEUS as a tool for therapy monitoring, further, larger studies are necessary, preferably according to a standardized examination protocol. In the future, a systematic follow-up of patients undergoing therapy using CEUS could lead to an early termination of ineffective therapy. In this way, patients could be offered an alternative. On the other hand, a less aggressive therapeutic approach could be made possible in patients with a very good response, with a corresponding reduction in side effects. Figure 2 shows a suggestion for possible examination dates.

Our own results indicate that multimodal ultrasound imaging is relevant. Metastatic lymph nodes are irregular, hardened in elastography, and show an irregular neovascularization in the marginal area as an early malignancy criterion [27]. Elastography, as well as CEUS, are becoming increasingly available in high-end devices and can be used for follow-up examinations and therapy response assessment.

A measurement after 2 weeks is used for early evaluation of the response. In the study by Chen et al., significant differences were already seen after this time. Studies investigating the monitoring of therapy by laboratory methods also show the importance of early evaluation [47]. Simultaneous performance with cross-sectional imaging after therapy is used to compare CEUS with the respective standard in follow-up imaging. By an additional measurement after half of the intended therapy, it can be further analyzed in which periods of the therapy a monitoring by CEUS is possible. 

## 5. Conclusions

Studies to date show the potential of CEUS to evaluate the therapeutic responses of metastatic lymph nodes even at an early stage. This could be a major advantage compared to other methods of therapy monitoring. As a result, ineffective therapeutic strategies could be detected quickly, non-invasively and cost-effectively. The importance of an appropriate therapy monitoring will continue to become more important in HNSCC along with the use of immunotherapeutic strategies. Before implementing this method in the therapy monitoring of solid tumors, further studies with large patient collectives and comparable examination protocols are necessary.

## Figures and Tables

**Table 1:** Inclusion and exclusion criteria according to the PICOS acronym.

	Inclusion Criteria	Exclusion Criteria
P (participants)	- Patients with a solid carcinoma - At least suspected lymph node metastasis based on imaging - Primary non-surgical treatment	- Haemato-oncological diseases - Mesenchymal malignancies - Primary surgical procedure with resection of the suspicious lymph nodes - Ablative treatment (e.g., radiofrequency)
I (intervention)	- CEUS prior to, during or after treatment - Examination of the metastatic lymph nodes using CEUS	- Examination of the primary tumor using CEUS - Examination of other metastases (e.g., liver)
C (comparisons)	not applicable	not applicable
O (outcomes)	- Analysis of qualitative and/or quantitative CEUS parameters (e.g., time to peak or area under the curve) - Evaluation of the therapy response of the patients	
S (study design)	- Original article	- Overview article - Reviews - Opinions/Comments - Conference Paper - Case reports

**Table 2:** Overview about the conditions of the included studies. *: 74 patients were included in the study, but only 64 completed both examinations and underwent surgery.

Reference	Publication Year	Study Design	Primary Disease	Cohort Size	Contrast Agent
Ye et al. [40]	2014	prospective	Nasopharyngeal carcinoma	67	SonoVue (2.4 mL)
Chen et al. [39]	2018	prospective	Nasopharyngeal carcinoma	20	SonoVue
Han et al. [41]	2021	prospective	Breast carcinoma	74/64 *	SonoVue (4 mL)

**Table 3:** Analyzed parameters and conclusions of the included studies. CEUS = contrast-enhanced ultrasound, PI = peak intensity, n.a. = data not available. *: Sensitivity and specificity for predicting therapeutic response of lymph nodes.

Reference	Qualitative Parameters Analyzed	Significant Qualitative Parameters	Quantitative Parameters Analyzed	Significant Quantitative Parameters	Sensitivity *	Specificity *	Conclusions
Ye et al. [40]			- Peak intensity (PI) - Time to peak	- PI and its change	- 72% (PI before therapy) - 94.3% (PI under ongoing therapy) - 92.5% (PI ratio)	- 52% (PI before therapy) - 88.2% (PI under ongoing therapy) - 83.8% (PI ratio)	- CEUS enables prediction of therapy success already in the early phase of radiochemotherapy
Chen et al. [39]			- Peak intensity (PI) - Area under the curve (AUC) - Time to PI - Mean transit time (mTT) - slope of wash-in - wash-in perfusion index	- Percentage changes in: - PI - Area under the curve - Slope of wash in - Wash in perfusion index	- n.a.	- n.a.	- CEUS is able to measure the effectiveness of therapy with famitinib at an early stage
Han et al. [41]	- uptake of the contrast agent: hilar or marginal/mixed - pattern of the contrast agent: even or uneven enhancement	- uptake of the contrast agent			- 87% (after neoadjuvant chemotherapy)	- 55.6% (after neoadjuvant chemotherapy)	- CEUS offers encouraging potential for predicting the success of neoadjuvant therapy in breast cancer

## Data Availability

No new data were created or analyzed in this study. Data sharing is not applicable to this article.
